# Abdominal symptoms in cystic fibrosis and their relation to genotype, history, clinical and laboratory findings

**DOI:** 10.1371/journal.pone.0174463

**Published:** 2017-05-04

**Authors:** Harold Tabori, Christin Arnold, Anke Jaudszus, Hans-Joachim Mentzel, Diane M. Renz, Steffen Reinsch, Michael Lorenz, Ruth Michl, Andrea Gerber, Thomas Lehmann, Jochen G. Mainz

**Affiliations:** 1 Jena University Hospital, Cystic Fibrosis Center, Jena, Germany; 2 Jena University Hospital, Pediatric Radiology, Jena, Germany; 3 Jena University Hospital, Pediatric Gastroenterology, Jena, Germany; 4 Jena University Hospital, Institute of Medical Statistics, Jena, Germany; University Children's Hospital Bern, SWITZERLAND

## Abstract

**Background & aims:**

Abdominal symptoms (AS) are a hallmark of the multiorgan-disease cystic fibrosis (CF). However, the abdominal involvement in CF is insufficiently understood and, compared to the pulmonary manifestation, still receives little scientific attention. Aims were to assess and quantify AS and to relate them to laboratory parameters, clinical findings, and medical history.

**Methods:**

A total of 131 patients with CF of all ages were assessed with a new CF-specific questionnaire (JenAbdomen-CF score 1.0) on abdominal pain and non-pain symptoms, disorders of appetite, eating, and bowel movements as well as symptom-related quality of life. Results were metrically dimensioned and related to abdominal manifestations, history of surgery, *P*. *aeruginosa* and *S*. *aureus* colonization, genotype, liver enzymes, antibiotic therapy, lung function, and nutritional status.

**Results:**

AS during the preceding 3 months were reported by all of our patients. Most common were lack of appetite (130/131) and loss of taste (119/131) followed by abdominal pain (104/131), flatulence (102/131), and distention (83/131). Significantly increased AS were found in patients with history of rectal prolapse (p = 0.013), distal intestinal obstruction syndrome (p = 0.013), laparotomy (p = 0.022), meconium ileus (p = 0.037), pancreas insufficiency (p = 0.042), or small bowel resection (p = 0.048) as well as in patients who have been intermittently colonized with *P*. *aeruginosa* (p = 0.006) compared to patients without history of these events. In contrast, no statistically significant associations were found to CF-associated liver disease, chronic pathogen colonization, lung function, CF-related diabetes, and nutritional status.

**Conclusion:**

As the complex abdominal involvement in CF is still not fully understood, the assessment of the common AS is of major interest. In this regard, symptom questionnaires like the herein presented are meaningful and practical tools facilitating a wider understanding of the abdominal symptoms in CF. Furthermore, they render to evaluate possible abdominal effects of novel modulators of the underlying cystic fibrosis transmembrane (conductance) regulator (CFTR) defect.

## Introduction

**Fig 1:**
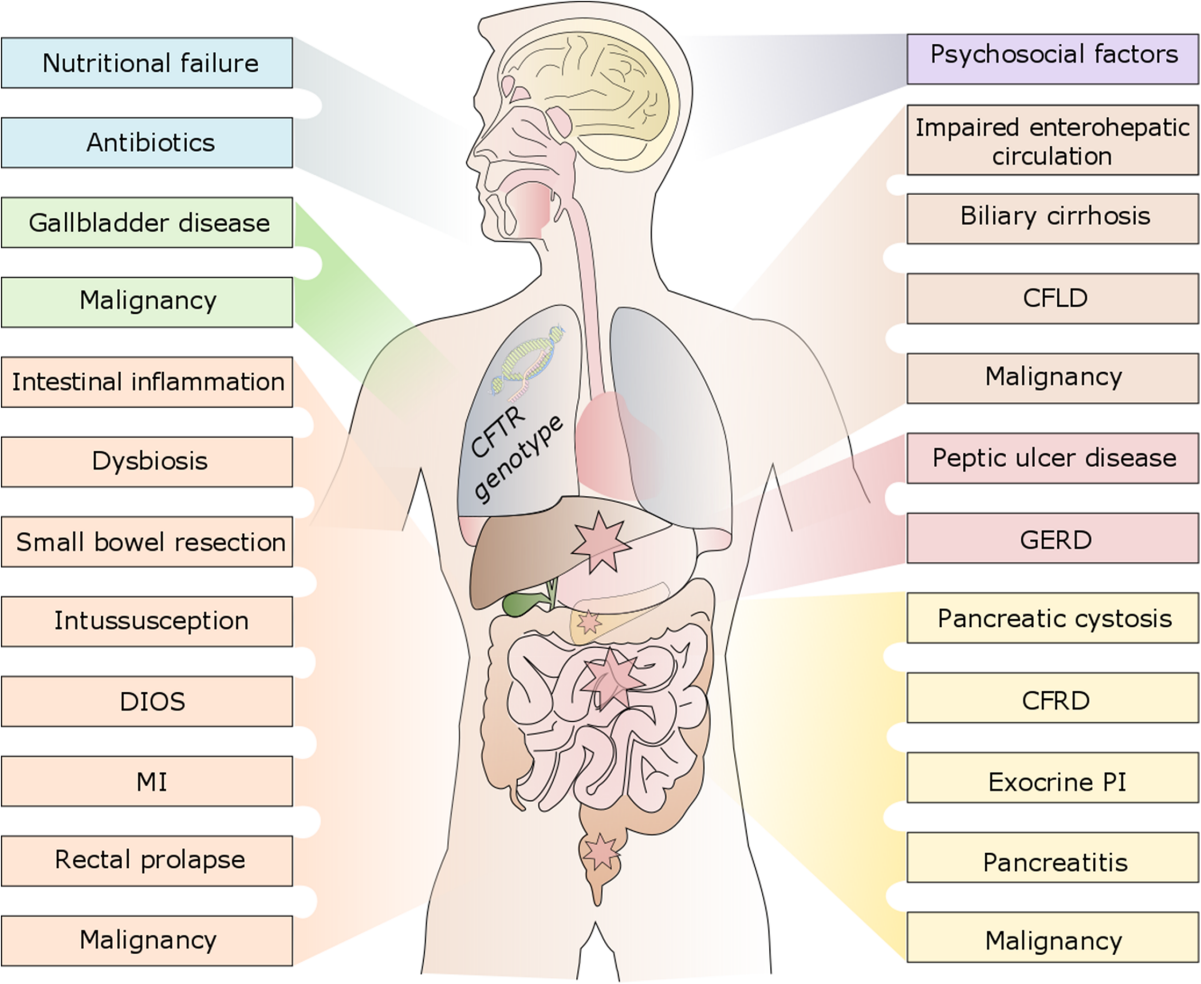
Multifactorial causes of abdominal symptoms in CF. CFLD–CF-associated liver disease, CFRD–CF-related diabetes, DIOS–distal intestinal obstruction syndrome, GERD–gastroesophageal reflux disease, MI–meconium ileus, PI–pancreatic insufficiency.

Cystic fibrosis (CF) is the most common life threatening autosomal recessive disorder caused by mutations in the cystic fibrosis transmembrane (conductance) regulator (*CFTR*) gene. The CFTR protein, which is essential in the regulation of chloride and sodium transport in epithelial cells [1], is highly expressed on the apical surface of intestinal epithelial cells, pancreatic ductal cells, and cholangiocytes in bile ducts which in the healthy transport ions, bicarbonate and fluid to the organs' lumen [2]. Thus, CFTR dysfunction results in viscous luminal secretions obstructing the bile and pancreatic ducts as well as the intestine [3]. The resulting gastrointestinal (GI) manifestations include pancreatic insufficiency (PI), meconium ileus (MI), distal intestinal obstruction syndrome (DIOS), and biliary tract complications which can lead to cirrhosis and hepatic failure [4]. Typical resulting GI symptoms are frequent and voluminous greasy stools, flatulence, abdominal bloating, constipation, abdominal pain, an impaired nutritional status, as well as failure to thrive. Even though GI symptoms are a hallmark of CF [5–7], often leading to diagnosis of the inherited disease, they are still insufficiently understood for why deeper investigations into the abdominal involvement in CF are needed [2]. Moreover, with enhanced survival due to improved therapeutic options and patient management, comorbidities of the GI, hepatobiliary, and pancreatic tract are of increasing clinical and scientific interest. However, because of the complex interaction of a variety of dysfunctioning organs, medicinal effects, and even psychosocial factors (Fig 1), the differentiation of the multitude of abdominal symptoms constitutes a major challenge.

Interestingly, previous studies assessing the general symptom of pain in CF indicate that its most common location was the abdomen [8–11]. Nevertheless, most of these studies did not differentiate abdominal pain e.g. regarding frequency, intensity and location, and thus failed to specify the origin of GI symptomatology and its clinical associations. In a recent systematic review, 16 studies investigating pain in CF were evaluated [12]. Eight of them reported occurrences of abdominal pain in CF, with a high variance ranging between 21% and 60% of the assessed CF patients, while only few studies measured pain intensity. This mostly was not specific for the abdominal region and based on single items scales (e.g. a numerical rating scale). It has been recognized that abdominal symptoms relevantly impair health related quality of life (HRQoL) by affecting CF patients' daily activities as well as their emotional, social and physical functioning [13]. Yet, only one study focused on reporting prevalence of recurrent abdominal pain in CF [14].

Recent developments of small molecules that potentiate or correct defective CFTR protein function entail a need to assess changes in abdominal involvement by the new systemic treatment. Most interestingly, in some mutations (e.g. *G551D*) CFTR modulators even allow restoration of pancreatic function in some patients [15,16] and they procured a trend towards normalization of sweat tests [17,18]. Besides outcome measures acquired by laboratory, radiological and electrophysiological methods, the Food and Drug Administration (FDA) encourages the usage of patient reported outcome measures (PROM) such as symptom questionnaires as supportive tools or even endpoint measures in clinical trials and offers guidance for their development [19].

Aim of the present study was to obtain structured and detailed information on GI involvement and symptoms with a new pilot score specifically designed for assessment of abdominal involvement in CF patients (JenAbdomen-CF Score 1.0) and relate results to clinical and laboratory findings, history, and *CFTR* genotype.

## Materials and methods

### Ethical statement

This study has been conducted in strict accordance with the ethical guidelines in the Declaration of Helsinki and it was approved by the Jena University ethics committee (registration number 4458-06/15). All patients aged ≥18 y and parents of minors provided written informed consent.

### Patients

The prospective study was performed including CF patients of all ages at the Jena University Hospital CF Center. Inclusion criteria were: (1) a diagnosis of CF determined by a sweat chloride of >60 mEq/L and/or (2) detection of 2 disease causing *CFTR* mutations with evidence of organ involvement.

### Evaluation of the score

**Fig 2:**
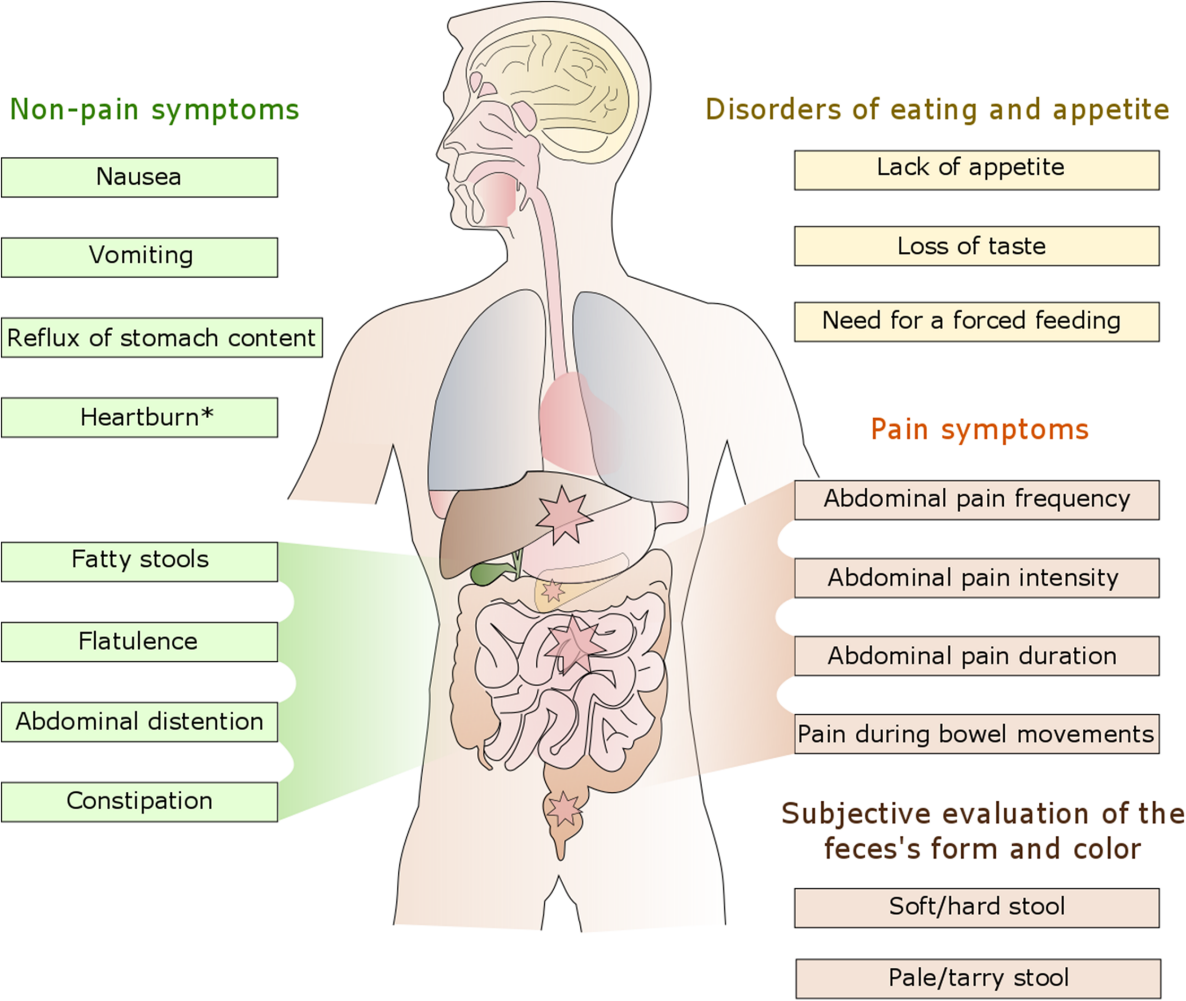
Pain and non-pain symptoms of the JenAbdomen-CF Score 1.0. *to some extend additionally related to pain symptoms.

The novel JenAbdomen-CF Score 1.0 was designed considering the recommendations given by the FDA for development of a PROM [19] including in-depth interviews with patients, literature reviews, and physician expert opinions. During routine presentation in our outpatient clinic, patients and/or the guardians completed a questionnaire consisting of 17 items (Fig 2, S1 Table) to measure the GI symptoms during the previous three months grouped into the following four domains:

1. abdominal pain,
2. non-pain symptoms,
3. subjective evaluation of the feces' frequency, form and color, and
4. disorders of eating and appetite.

1. The abdominal pain domain consists of three items which assess frequency, intensity, and duration of abdominal pain. In addition, one item regarding the intensity of pain during bowel movements was evaluated. Pain frequency was measured using a Likert-type scale [20], with six response options ranging from `never´ (0pts) to `daily´ (5pts). Pain intensity was assessed using a well-validated Visual Analogue Scale (VAS) [21], which consists of an 11-point scale ranging from 0 to 10 with a series of six emotion expressing faces anchored at either end by `no pain´ (0pts) to `worst pain ever´ (5pts; 2 VAS steps each). The duration of experienced abdominal pain was assessed by offering six options ranging from `0 minutes´ (0pts) to `more than 360 minutes´ (5pts). In addition, we asked for the location, aggravating and alleviating factors, coping strategies, radiation, onset and quality of abdominal pain. Location of the abdominal pain was marked on a well-validated body outline adapted from Savedra et al. [22] displaying an anterior and posterior view of the abdomen. Coding of body location was conducted using the nine quadrants of abdomen including: hypochondriac (right and left); epigastric; lumbar (right and left); umbilical; iliac (right and left) and hypogastric regions. Cut-off points for mild (VAS <3), moderate (VAS 3–5) and severe (VAS ≥5) abdominal pain was given according to Kainzwaldner et al. [23].
2. The non-pain symptoms consist of 8 items which include flatulence, abdominal distention, constipation, nausea, vomiting, heartburn, fatty stools, and reflux of stomach content. Each symptom was measured with a 6-point rating scale anchored at either end by `not at all´ (0pts) to `always´ (5pts) at the other end, except for fatty stools that was assessed by a binary response: `no´ (0pts) and `yes´ (5pts).
3. The consistency of stool (one item) was evaluated using an adaptation of the well-validated Bristol Stool Form Scale [24,25] classified into seven types (type 1–2 = hard; type 3–4 = formed; type 5–7 = soft). Zero points were given for this item by a formed stool consistency; 1 point for a hard stool consistency; 3 points for both hard and soft stool consistency and 5 points for a soft stool consistency. The patient’s stool color (one item) was assessed using a modified Stool Color Card for the screening of biliary atresia by addition of five from brown to black, tarry stool colors adapted from Gu et al. [26] with 12 consecutive pictures ranging from pale to tarry stools (1–3 = pale; 4–11 = normal; 12 = tarry). For this scoring, 0 points were given for a normal color, 5 points for a pale color and 3 points for a tarry color.
4. Disorders of eating and appetite were assessed questioning the following three items: lack of appetite, loss of taste, and need for a forced feeding (by the patient himself or by others). The first two items were measured on a 6-point rating scale anchored at either end by `not at all´ (0pts) to `always´ (5pts), respectively. The last item was evaluated by a binary response: `no´ (0pts) and `yes´ (5pts). A total of 17 items were evaluated so that the sum of obtained points could range from 0 to 85 points with higher rates for increasing severity of GI symptoms. Abdomen scores were compared with each other and gastrointestinal CF manifestations, history of surgery, the nutritional status, *CFTR* genotype, liver function test (LFT) including alanine aminotransferase (ALT), aspartate aminotransferase (AST), gamma-glutamyl transpeptidase (GGT), total bilirubin (BIL), and alkaline phosphatase (ALP), adherence to pancreas enzyme intake (self-reported), antibiotic therapy, weight (ΔWtP), height (ΔHtP) and BMI (ΔBMIP) percentiles changes in the previous 3, 12, and 24 months for patients <18 yrs and ΔBMI in the previous 3, 12, and 24 months in adults, lung function including FEV1%, and airway colonization with *P*. *aeruginosa* (PsA) and/or *S*. *aureus* (SA).

### Classification of *CFTR* genotype

The recently established pancreatic insufficiency prevalence (PIP) scores [27] were used to measure the severity of specific *CFTR* mutations in regard to pancreatic function. The PIP score is calculated as the proportion of PI among all patients (PI and non-PI) carrying the same CF-causing mutation in a homozygous or in a heterozygous state, in the latter case considering the lower PIP value for both alleles. The term `genotype´ therefore refers to the combination of *CFTR* mutations on both alleles, accounting for the milder of both alleles for characterization of the PIP score [28]. According to the Canadian Consortium for CF Genetic Studies (CCCFGS), CF mutations are classified as `mild´ regarding pancreatic involvement when PIP is ≤0.25 and as `moderate-severe´ when PIP is >0.25. The patients carrying at least one mutation not reported from Ooi that could not be attributed to a specific PIP score were excluded from the genotype analysis (15/131 patients in the Jena CF center).

### Measures of clinical data

Demographic, clinical, and laboratory data were obtained from the charts. Nutritional failure was defined according to the 2002 Cystic Fibrosis Foundation (CFF) criteria [29]. Specifically, weight-for-height percentile (WHp) <10^th^ for ages 0–2 y, or body mass index percentile (BMIp) <10^th^ for ages 2–20 y were used to identify underweight. BMI was calculated as [weight in kilograms/(height in meters)^2^]. Age- and gender-specific percentiles for BMI (BMIp), weight (Wtp) and height (Htp) were classified according to the longitudinal local anthropomorphic data from Jena obtained by Krohmeyer-Hauschild [30]. Changes in Wtp, Htp and BMIp were calculated by subtracting baseline from values in the previous 3, 12, and 24 months, thus a negative value corresponded to a decrease in Wtp, Htp, and BMIp and a positive value to an increase of those. A potentially clinically significant (PCS) LFT elevation was defined as ALT/AST/GGT >3× upper limit of normal (ULN) or BIL/ALP >2× ULN. CF-associated liver disease (CFLD) was defined according to recently published guidelines for the diagnosis and management of CFLD [31]. Pulmonary disease severity was divided into three groups accounting FEV1 ≥70 percent of predicted (pp) as `mild´ disease, FEV1 40–69 pp as `moderate´ disease, and FEV1 ≤39 pp as `severely advanced´ lung disease. This established classification has been adopted internationally as a categorization of disease severity for CF [32–34]. Status of PsA and SA colonization were defined according to Leeds criteria [35].

### Data analysis

Statistical analyses were performed with SPSS, Version 23.0 (IBM Corp. 2015, Version 23.0. Inc., Armonk, NY, USA). Normal distribution of the data was tested using the Kolmogorov-Smirnov (K-S) test. Parametric tests were used to compare means between two independent samples (two-tailed Student t-Test) or more than two groups (ANOVA), when the samples were normally distributed. When criteria for normal distribution were not met, nonparametric tests were chosen to detect statistical difference in means of two independent samples (Mann-Whitney U test) or more than two groups (Kruskal-Wallis test). Nominal data were compared with Chi-square test or Fisher’s exact tests, as appropriate. Correlations between variables were examined using the Pearson’s correlation coefficient, the Spearman’s rank correlation coefficient and the mean square contingency coefficient, as appropriate. Data are given as means ± SD. A p-value ≤0.05 indicated a significant difference or correlation.

## Results

### Baseline characteristics of the total study cohort

Between April 2015 and December 2015 a total of 131 CF patients attended in the Jena University CF center were included into the prospective study. The mean age was 19.1±14.2 years. Mean FEV1 was 84%predicted and 31 patients (24%) had CF-related diabetes, of whom 14 (45%) were insulin dependent. CF diagnosis most frequently was established on the basis of either predominant GI or respiratory symptoms, whereby 24% of the patients presented a combination of symptoms. *CFTR* gene mutations were identified on both alleles in all patients. The most common *CFTR* mutation in the German population, *F508del*, was detected in 117/131 (89%) of the included CF patients, with 56 (43%) being homozygous for this *CFTR* defect. *G551D* was detected in 17 patients (13%). Further characteristics of CF patients are presented in Table 1 and in S1 Table.

**Table 1:** Patient characteristics.

Variable	Frequency (n = 131)	
Gender		
Male	58/131	(44.3%)
Female	73/131	(55.7%)
*CFTR* genotype		
*F508del/ F508del*	56/131	(42.7%)
*F508del/ other*	61/131	(46.6%)
*G551D/ other*	17/131	(13.0%)
*ther/ other*	14/131	(10.7%)
Age (y)		
0–5	23/131	(17.6%)
6–11	27/131	(20.6%)
12–17	24/131	(18.3%)
≥18	57/131	(43.5%)
Nutritional status (<18 yrs.)		
Underweight	6/74	(8.1%)
Short stature	2/74	(2.7%)
*P*. *aeruginosa* (PsA): chronic	41/129	(31.3%)
*P*. *aeruginosa* (PsA): intermittent	16/129	(12.2%)
Antibiotic therapy		
Intravenous therapy	27/131	(20.6%)
Oral therapy	85/131	(64.9%)
Inhaled therapy	55/131	(42.0%)
Abdominal surgeries		
Laparotomy	17/131	(13.0%)
Small bowel resection	12/131	(9.2%)
Appendectomy	12/131	(9.2%)
Elevated liver function test (LFT)	90/129	(69.8%)
CF abdominal manifestations		
Exocrine pancreatic insufficiency (PI)	121/131	(92.4%)
PIP score `mild´	4/116	(3.4%)
PIP score `moderate-severe´	112/116	(96.6%)
Meconium ileus (MI)	12/131	(9.2%)
Distal intestinal obstruction syndrom (DIOS)	11/131	(8.4%)
Rectal prolapse	14/131	(10.7%)
CF-associated liver disease (CFLD)	25/122	(20.5%)
CF-related diabetes (CFRD)	31/131	(23.7%)

### JenAbdomen-CF Score 1.0

#### Abdominal symptoms

**Fig 3:**
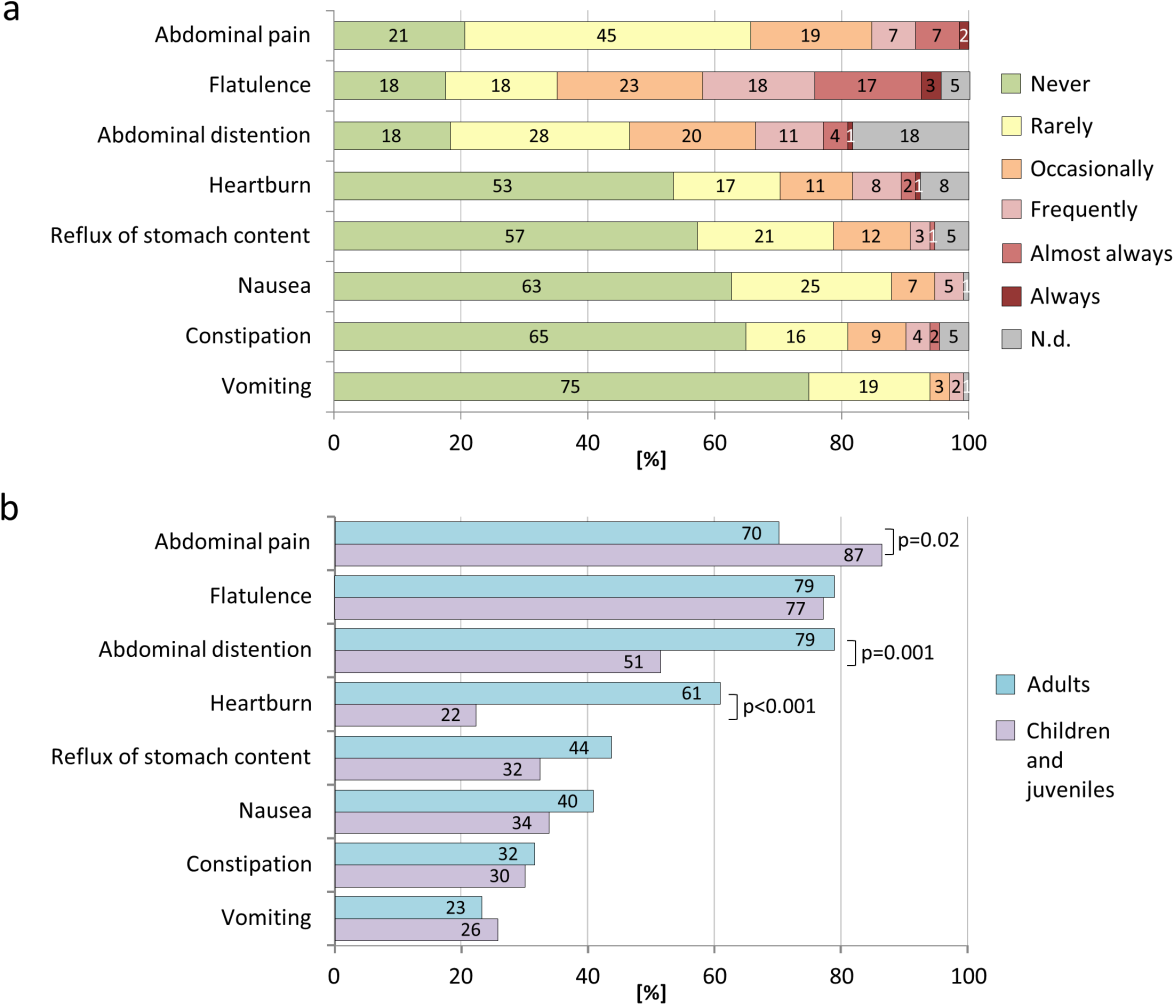
Abdominal symptoms in patients with CF. Frequencies of abdominal symptoms in CF patients of all ages (Fig 3a) and in children compared with adults (Fig 3b). N.d.–not determined (missing data).

Abdominal symptoms during the previous three months were reported from all CF patients. Most common were lack of appetite (99%) and loss of taste (91%; Table 2) followed by abdominal pain (79%), flatulence (78%), and abdominal distention (63%; Fig 3A, Table 2). Interestingly, children reported to have more abdominal pain than adults (87% vs. 70%; p = 0.022), while adults more frequently reported abdominal distention (79% vs. 51%; p = 0.001) and heartburn (61% vs. 22%; p<0.001; Fig 3B). 11% of patients admitted they forgot to take pancreas enzyme at least once a week. These patients did not report significantly more GI symptoms than patients who had good adherence to treatment (21.7 vs. 19.6 score points; p = 0.280).

**Table 2:** Frequency of abdominal symptoms in patients with CF.

Abdominal symptoms (Item)	Responded [%]	Reported symptom^a^ [n]	Mean symptom score^b^ (SEM)
Lack of appetite	99.2	130	2.1 (0.1)
Loss of taste	90.8	119	1.6 (0.1)
Abdominal pain	100	104	−− ^c^
Flatulence	95.4	102	2.6 (0.1)
Abdominal distention	81.7	83	1.9 (0.1)
Pain during bowel movements	96.2	58	1.5 (0.1)
Fatty stools	97.0	57	−− ^d^
Soft and hard stool consistency	98.5	54	−− ^e^
Heartburn	92.4	51	1.9 (0.1)
Reflux of stomach content	94.7	49	1.6 (0.1)
Nausea	99.2	48	1.4 (0.1)
Constipation	95.4	40	1.7 (0.1)
Vomiting	99.2	32	1.3 (0.1)
Forced feeding	99.2	20	−− ^d^
Soft stool consistency	98.5	12	−− ^e^
Pale stool	98.5	6	−− ^f^
Hard stool consistency	98.5	2	−− ^e^
Tarry stool	98.5	2	−− ^f^

^a^Item scale `1–5´ (anything but `0´)

^b^within the respective item (item scale `1–5´)

^c^response type: Likert

^d^binary response type

^e^Bristol Stool Form Scale

^f^Stool Card Color

#### Abdominal pain

**Fig 4:**
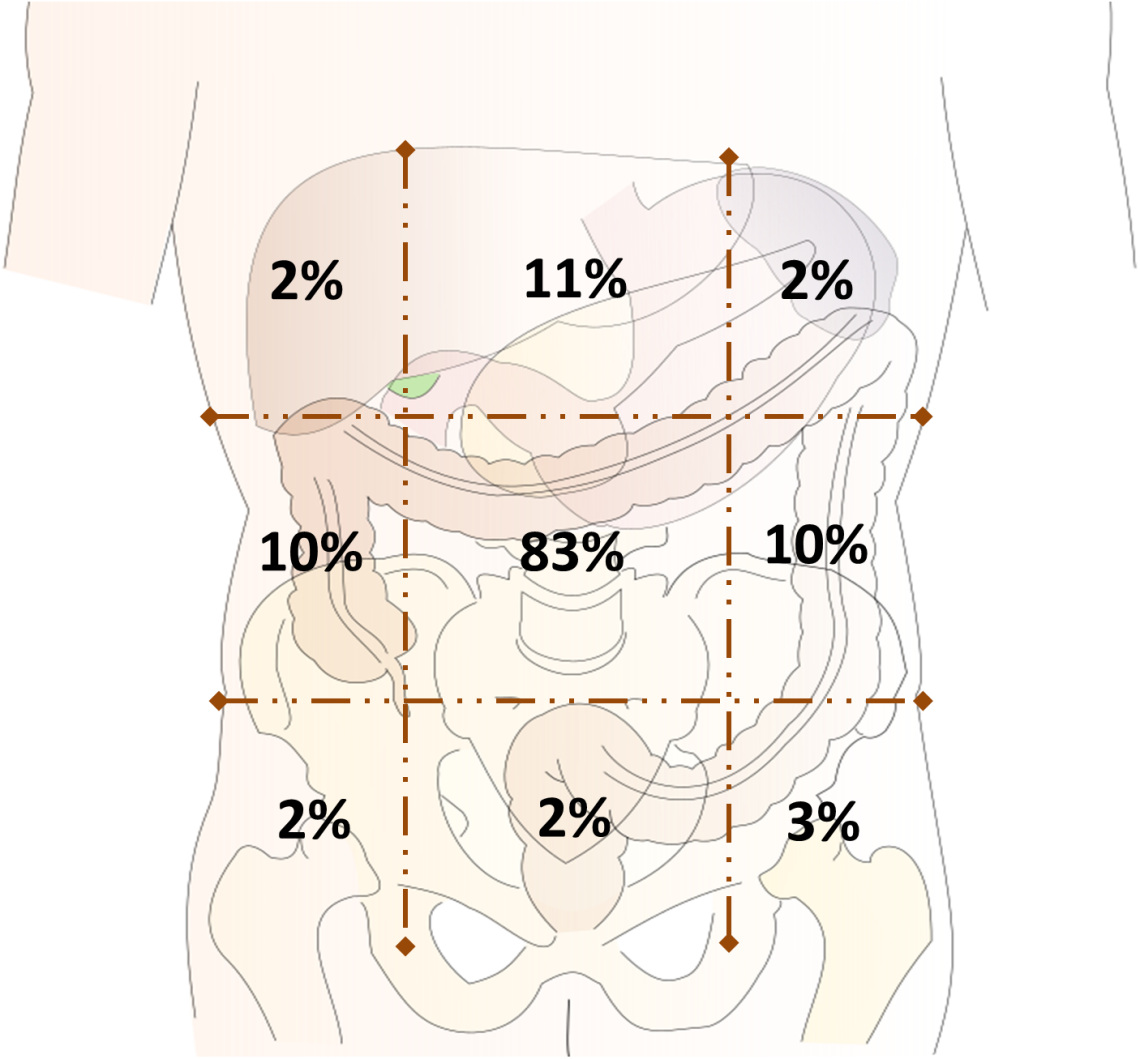
Location of abdominal pain in patients with CF.

The most frequent locations of abdominal pain were the umbilical (83%) and epigastric regions (11%), and 8% of patients reported a radiation of pain to the dorsum (Fig 4). More than one pain location was reported by 28%. Pain intensity on the visual analogue scale (VAS) resulted in a mean of 3.4 of maximally 10 points (SD: 2.3). Of these patients, 7% had mild- (VAS: 1-3pts), 43% moderate- (VAS: 4-5pts), and 30% severe pain (VAS: ≥5pts). Thereby, 34% reported a frequency of abdominal pain occurring `at least once a week´ (Table 3). Most common quality of abdominal pain experienced were `pulling´ (42%), `colicky´ (41%) and `sharp´ (28%). 49% of patients described abdominal pain lasting `less than 45 minutes´ and a small subgroup of patients (8%) lasting `five hours´ or `longer´. It appeared more frequently `during meals´ (35%), `before bowel movements´ (34%), and `during stressful events´ (12%). Interestingly, a small subgroup of patients (3%) reported that pain is relieved `after antibiotics administration´ and 10% reported amelioration by `bowel movements´.

**Table 3:** Abdominal pain characteristics.

Characteristic	Total (n = 131)		Children (n = 74)		Adults (n = 57)	
Intensity (VAS)	3.4 ± 2.3		3.7 ± 2.1		3.1 ± 2.5	
Frequency						
Never	27/131	(20.6%)	10/74	(13.5%)	17/57	(29.8%)
Ca. once a month	59/131	(45.0%)	29/74	(39.2%)	30/57	(52.6%)
Ca. once a week	25/131	(19.1%)	21/74	(28.4%)	4/57	(7.0%)
Each 2–4 days	9/131	(6.9%)	6/74	(8.1%)	3/57	(5.3%)
Almost daily	9/131	(6.9%)	6/74	(8.1%)	3/57	(5.3%)
Daily	2/131	(1.5%)	2/74	(2.7%)	0/57	(0.0%)
Location						
Umbilical	77/93	(82.8%)	48/54	(88.9%)	29/39	(74.4%)
Right hypochondriac	2/93	(2.2%)	1/54	(1.9%)	1/39	(2.6%)
Epigastric	10/93	(10.8%)	6/54	(11.1%)	4/39	(10.3%)
Left hypochondriac	2/93	(2.2%)	1/54	(1.9%)	1/39	(2.6%)
Left lumbar	9/93	(9.7%)	4/54	(7.4%)	5/39	(12.8%)
Right lumbar	9/93	(9.7%)	1/54	(1.9%)	8/39	(20.5%)
Left iliac	3/93	(3.2%)	0/54	(0.0%)	3/39	(7.7%)
Hypogastric	9/93	(9.7%)	3/54	(5.6%)	6/39	(15.4%)
Right iliac	9/93	(9.7%)	2/54	(3.7%)	7/39	(17.9%)
Quality						
Pulling	35/83	(42.2%)	19/44	(43.2%)	16/39	(41.0%)
Colicky	34/83	(41.0%)	17/44	(38.6%)	17/39	(43.6%)
Sharp	23/83	(27.7%)	11/44	(25.0%)	12/39	(30.8%)
Burning	4/83	(4.8%)	1/44	(2.3%)	3/39	(7.7%)
Pressing	3/83	(3.6%)	2/44	(4.5%)	1/39	(2.6%)
Crampy	2/83	(2.4%)	1/44	(2.3%)	1/39	(2.6%)
Duration (min)						
0	26/108	(24.1%)	10/58	(17.2%)	16/50	(32.0%)
<45	53/108	(49.1%)	37/58	(63.8%)	16/50	(32.0%)
45–90	6/108	(5.6%)	4/58	(6.9%)	2/50	(4.0%)
91–180	11/108	(10.2%)	2/58	(3.4%)	9/50	(18.0%)
181–360	3/108	(2.8%)	2/58	(3.4%)	1/50	(2.0%)
>360	9/108	(8.3%)	3/58	(5.2%)	6/50	(12.0%)
Onset						
Suddenly	50/81	(61.7%)	27/44	(61.4%)	23/37	(62.2%)
Progressively	31/81	(38.3%)	17/44	(38.6%)	14/37	(37.8%)
Radiation to dorsum	7/90	(7.7%)	5/52	(9.6%)	2/38	(5.3%)

VAS: visual analogue scale

**Fig 5:**
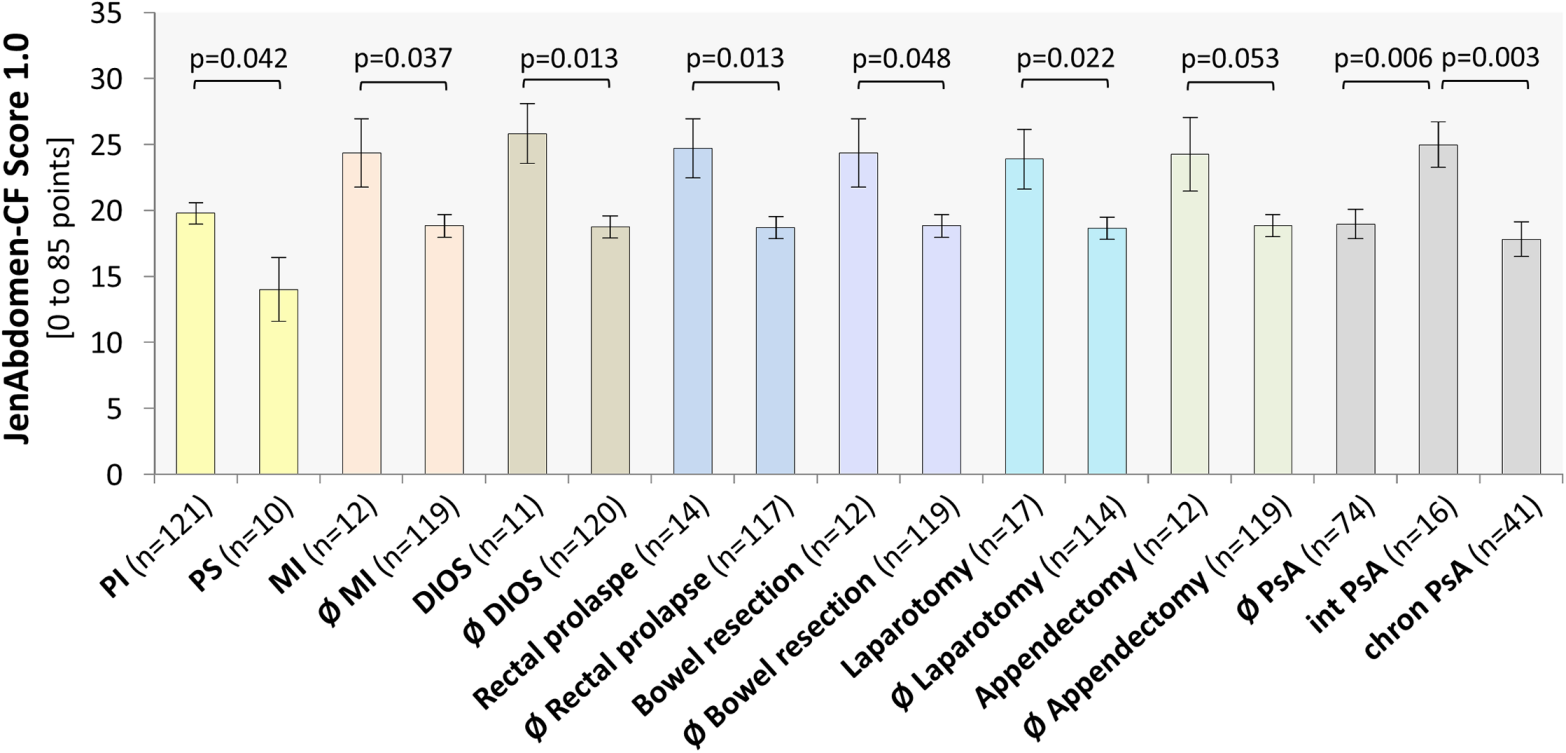
JenAbdomen-CF Score 1.0 of patients presenting indicated abdominal manifestations and complications of surgery in comparison with patients without history of these events (Ø). Int PsA–intermittently colonized with *P*. *aeruginosa*, chr PsA–chronically colonized with *P*. *aeruginosa*. Means ± SEM.

The percentage of missing item responses on the abdomen score was 4.8%. Abdomen scores were non-normally distributed (different sizes of subgroups, e.g., PI and PS) with a mean/median (range) of 19.3/18.0 (3–46). Altogether, female patients revealed slightly higher abdomen scores than males (20.4 vs. 18.0; p = 0.139). Moreover, no significant difference was observed among age subgroups (<18 y: 19.7 vs. ≥18 y: 18.9; p = 0.385). In contrast, significantly increased AS were found in patients with history of rectal prolapse (p = 0.013), distal intestinal obstruction syndrome (p = 0.013), laparotomy (p = 0.022), meconium ileus (p = 0.037), pancreatic insufficiency (p = 0.042), or small bowel resection (p = 0.048) as well as in patients who have been intermittently colonized with PsA (p = 0.006) compared to patients without history of these events (Fig 5). For appendectomy, a strong trend was seen (p = 0.053; Fig 5).

#### *CFTR* genotype

**Fig 6:**
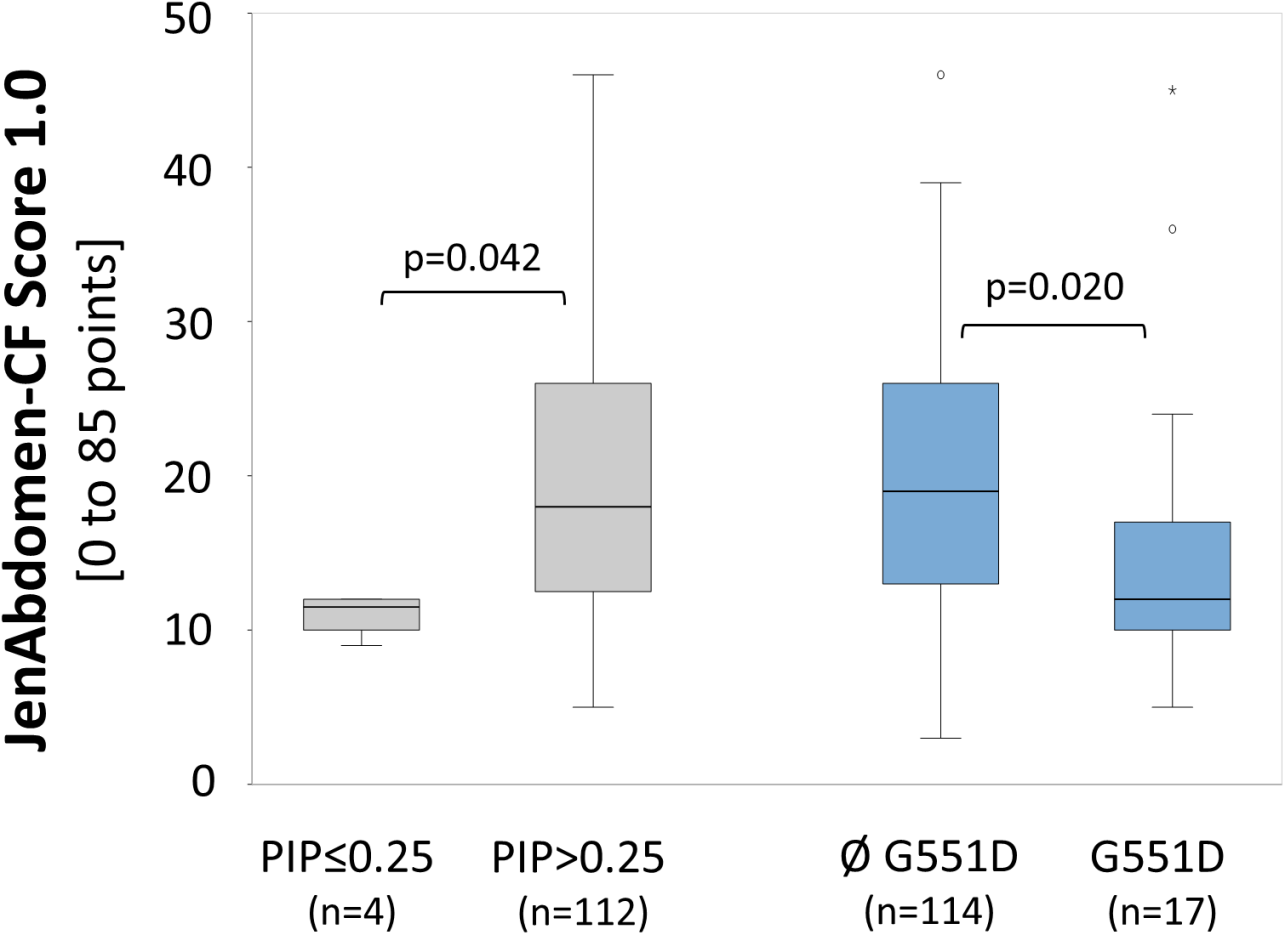
JenAbdomen-CF Score 1.0 in relation to genotype. Left: Scores obtained from `mild´ (PIP≤0.25) compared to `moderate-severe´ (PIP>0.25) genotypes. Right: Scores of patients carrying the *G551D* mutation compared to those without this mutation. Of note, the fact that 10/17 of the patients with *G551D* mutation received ivacaftor may have influenced the result.

Patients who carry mild genotypes (PIP score ≤0.25) had significantly lower rates for GI symptoms compared to those with severe mutations (PIP score >0.25) (11.0 vs. 19.6; p = 0.042). In addition, patients with a *G551D* mutation on at least one allele had significantly lower JenAbdomen-CF Scores 1.0 compared to patients without this mutation (15.6 vs. 19.9; p = 0.020). Of these patients, 59% (10/17) were treated with ivacaftor (Fig 6).

#### CF-associated liver disease (CFLD)

A total of 122 (93%) patients were assessed for a diagnosis of CFLD. Patients who underwent liver transplantation (n = 2) or had <2 consecutive examinations spanning a 1-year period (n = 7) were excluded from the evaluation of a liver disease. Twenty-five of 122 patients (21%) met the criteria for CFLD and seven of these had liver cirrhosis (6% of all included patients). There were no significant differences between CFLD patients and those without liver involvement concerning the JenAbdomen-CF Score 1.0 (18.3 vs. 19.8; p = 0.487). Interestingly, JenAbdomen-CF Score 1.0 did not differ significantly between non-cirrhotic CFLD individuals and CF patients without liver involvement (17.9 vs. 19.8; p = 0.462). Even in cirrhotic CFLD individuals, JenAbdomen-CF Score 1.0 did not reveal a significant difference when compared to patients without liver involvement (20.0 vs. 19.8; p = 833).

#### Liver enzymes in serum

Of the 131 patients, 129 were tested for liver enzymes in the past three months. A total of 90/129 participants (70%) had at least one elevation above the ULN and 14% (18/129) presented elevations of LFT considered as PCS. There was no statistically significant difference in mean JenAbdomen-CF Scores 1.0 between patients with and without elevated LFT (19.6 vs. 18.9; p = 0.699). Similarly, there was no difference between LFT considered as PCS and those without PCS consideration (19.6 vs. 19.3; p = 0.897).

#### Nutritional status

No significant differences in JenAbdomen-CF Score 1.0 were observed between CF patients with a reduced (nutritional failure) and stable nutritional status. Additionally, there were no significant differences in JenAbdomen-CF Score 1.0 between negative and positive changes in Wt, Ht and BMI in the previous 3, 12, and 24 months (data not shown).

#### Lung function

Patients with `severe´ lung disease showed slightly decreased scores compared to patients with `moderate´ lung disease (17.2 vs. 19.7; p = 0.360) and `mild´ lung disease (17.2 vs. 19.5; p = 0.402).

#### Antibiotics

Of all included patients, 97 (75%) were treated with oral, intravenous, or inhaled antibiotics during the past three months. Average JenAbdomen-CF Scores 1.0 were slightly higher in children who received any antibiotics therapy compared with adults treated with antibiotics (20.5 vs. 18.9; p = 0.175). Among all patients who received antibiotics, patients treated with ciprofloxacin and/or meropenem tended to score higher than patients treated with any other antibiotics (23.3 vs. 19.0; p = 0.085).

## Discussion

Although GI involvement is a hallmark of CF, until now it received comparatively little clinical and, even less, scientific attention. To our best knowledge, GI symptoms in CF as a multiorgan manifestation have not yet been systematically quantified. Here, we present data of a new questionnaire assessing the complexity of GI symptoms from 131 CF patients of all ages; the questionnaire prospectively shall be elaborated to a standardized and validated abdominal CF score, considering the FDA guidelines [19]. Additionally, quantified symptoms were related to phenotypic characteristics and to laboratory findings. The JenAbdomen-CF Score 1.0 questionnaire is a simple but meaningful two-page instrument capable to detect differences between several CF phenotypic characteristics, as shown within this publication. For purposes of monitoring, it can quickly and therefore routinely filled-in at on-site visits. In clinical trials, it can be implemented as an additional easy and inexpensive tool of, however, high relevance.

First of all, our study showed that all included CF patients presented at least one abdominal symptom within the preceding three months. A high prevalence rate of GI symptoms in 60% of 70 CF patients has also been reported in a recent survey by Fraquelli et al. [36]. This study, however, considered a lower number of items for the evaluation of the GI symptoms (7 items, compared to 17 items in the JenAbdomen-CF Score 1.0) what might explain the difference in the prevalence rates. Nevertheless, high prevalence rates of GI symptoms are known to be representative in CF. For reasons of comparison, clear distinction, and specification, age-matched healthy controls should be included in future research on CF-related GI symptoms using the questionnaire.

In our study, around one-third (34%) of CF patients experienced at least one episode of abdominal pain per week with a small subgroup suffering `almost always´ (7%) and `always´ (2%) from the relevant symptom. By comparison, Munck et al. [14] reported a very low prevalence of recurrent abdominal pain (RAP) of 6%, yet using the more strict Apley’s criteria (at least three bouts of pain, severe enough to affect one´s activities, over a period of not less than three months, with attacks continuing in the year preceding the examination) [37]. In our study, the mean abdominal pain intensity was indicated as moderate (3 VAS pts; median = 4/max = 10) and of relatively short duration (<45 min), which is in the range of previous reports [8,10]. The majority of studies on abdominal pain in CF, however, did not specify the localization of pain [8,10,38,39].

In the 85% of patients with exocrine PI, enzyme substitution may relieve many but not all GI symptoms. Thereby, low treatment adherence is considered a major cause of increased symptomatology. That adherence to pancreatic enzyme intake was self-reported may constitute a limitation of our study, as maladherence might be under-reported. Of all included PI patients, 76% stated to have taken their enzymes regularly (never or less than once a month missed a dose). Furthermore, 3% stated to have forgotten them several times a week, 7% about once a week, and 14% several times a month. This self-reported rate of adherence regarding enzyme intake is comparatively high. For instance, Modi et al. only estimated an adherence rate at 50% or below [40]. Compared to objective measures, self-reports in general involve some uncertainty and may be biased for several reasons. In consecutive studies it would be most interesting to use objective methods to measure adherence to enzyme intake and its correlation to symptoms but possibilities to effectively and objectively control intake of tablets within daily life are markedly limited.

Altogether, in our study, female subjects revealed slightly higher JenAbdomen-CF Score 1.0 values than males. To some extent, this may be explained by an overlap with some symptoms associated with the menstruation cycle. In accordance, significantly more abdominal pain has been described both in CF and in healthy pubescent girls [14,41]. Of note, our study revealed no differences in the overall abdomen scores between the age groups. This accords well to a recent systematic review showing that prevalence and intensity of nonspecific pain was not linked to age in CF [12].

Our results showed slightly higher abdomen scores in patients with mild lung symptomatology (FEV1 ≥70 pp; 19.5 score points) compared to patients with severe lung disease (FEV1 ≤39 pp; 17.2 score points). In line with this, it has recently been noticed that mild lung disease in CF is associated with more severe extrapulmonary manifestations [42].

In general, CFLD reveals a slow progression over years and even decades and often is asymptomatic until most advanced stages [31,43,44]. In accordance, we did not see differences in abdomen scores among non-CFLD, non-cirrhotic CFLD, and cirrhotic CFLD groups. Furthermore, six of the seven cirrhotic CFLD individuals included in our study were classified with Child-Pugh score A indicating compensated liver cirrhosis without signs of portal hypertension.

In our study, CF patients with nutritional failure (reduced nutritional state) did not report higher rates of GI symptoms. This finding can be explained by the retrospective and cross sectional character of our questionnaire. In a prospective study, Shoff et al. [45] reported that a better nutritional status was associated with increased HRQoL scores. This questionnaire, however, included only one item on `digestive symptoms´. Thus, further prospective studies using meaningful questionnaires are warranted to evaluate the impact of GI symptoms on the nutritional status of CF patients. In this respect, additional factors that contribute to growth failure such as intestinal inflammation [46–49] and dysbiosis [50,51] should be considered.

A main finding of our study was that the majority of patients who underwent laparotomy scored higher than those without such surgery. This might be attributed to a more severe course of gastrointestinal disease, causing volvulus, intestinal atresia, intussusception, MI, and DIOS in these patients. Interestingly, in 15 of these 17 patients, PIP scores were ≥0.96 that accords to PI in almost all of these patients.

Among children and adolescents, those who received several antibiotic i.v. courses tended to suffer from more pronounced GI symptoms than patients without antibiotic treatments. Especially early in life, antibiotic treatment may affect the intestinal microbiota with putative long-term health consequences [52]. The usage of broad-spectrum antibiotics has been suspected to increase the risk of abdominal pain on the one hand [52]. On the other hand, it has been associated with a reduction in physiological anaerobic species and enterobacteria in the intestinal flora [53,54]. Some studies have shown that ciprofloxacin particularly suppresses gram-negative rods such as *Enterobacteriaceae* and *Bacteroides* species [55,56], thus promoting dysbiosis in CF [50]. In accordance, we observed higher symptom scores in patients with intermittent *P*. *aeruginosa* colonization, who often receive longer courses of ciprofloxacin.

Altogether, the 17 CF patients of our cohort carrying at least one *G551D-CFTR* mutation showed lower scores than non-*G551D-CFTR* patients (13.6 vs. 17.0; p = 0.029). As 59% of these patients (10/17) received ivacaftor at the time of questioning, we cannot distinguish whether this outcome was due to therapy or because of the general milder clinical phenotype compared to *F508del*, as previously suggested [57,58]. In addition, patients treated with ivacaftor (IVA) reported significantly lower scores in comparison with non-*G551D-CFTR* patients (11.7 vs. 17.0; p = 0.032). In line with this, we herein propose the usage of our questionnaire with special emphasis on its value for future clinical studies. As CFTR modulators such as IVA act systemically, the assessment of changes in abdominal symptoms is of outstanding interest, especially, since first CFTR modulators for frequent mutations like *F508del* are now available [59], and many studies with novel modulating substances are on the way. Thereby, a deeper understanding of the complex abdominal involvement is needed and assessment of changes of abdominal symptoms during CFTR modulation is of high interest. In light of this, the complementing usage of PROMs such as symptom questionnaires like the herein presented is promising.

As mentioned above, one limitation of our study is its retrospective character. Furthermore, missing values in the questionnaire were replaced by zero what might have introduced biased estimates interfering with the final score. However, the percentage of missing data was only 4.8%, what can be considered as low, according to Monte Carlo [60].

Finally, from our data we can conclude that patients with a severe course of the disease or with genotypes causing a moderate to severe abdominal involvement (what at the same time more frequently is associated with history of DIOS, MI, PI and rectal prolapses) are more likely to have higher rates for GI symptoms. Concurrently, GI symptomatology was not associated with CFLD or elevated LFTs.

Meanwhile, we have elaborated a revised and improved version of our questionnaire and the deduced JenAbdomen-CF Score, which now will undergo the following steps:

- Evaluation of reliability of the questionnaire by examining internal consistency and construct validity,
- Evaluation of reproducibility of the questionnaire by re-testing of patients,
- Assessment of cross-generational applicability of the questionnaire by subscoring of age groups,
- Evaluation of the responsiveness of the score by comparison with age-matched healthy controls,
- Evaluation of the power of the questionnaire by comparison with the Cystic Fibrosis Questionnaire Revised (CFQ-R) which inquires mainly QoL items,
- Assessment of applicability of the questionnaire in other CF centers,
- Assessment of putative relationships of abdominal symptoms with faecal inflammatory markers.

Prospectively, we aim at disseminating and applying our JenAbdomen-CF Score to a larger patient cohort not only in Germany but also in other countries in order to assess practicability of the questionnaire and provide proof of principle for the significance of the thus calculated score on GI involvement in CF.

## Supporting information

S1 Table (XLSX)

## Abbreviations

CFTR: cystic fibrosis transmembrane (conductance) regulator
CF: cystic fibrosis
GI: gastrointestinal
PI: pancreatic insufficiency
PS: pancreatic sufficiency
DIOS: distal intestinal obstruction syndrome
MI: meconium ileus
CFLD: CF-associated liver disease
CFRD: CF-related diabetes
RP: rectal prolapse
PIP: pancreatic insufficiency prevalence
SA: *Staphylococcus aureus*
PsA: *Pseudomonas aeruginosa*
Wtp: weight percentile
Htp: height percentile
BMIp: body mass index percentile
LFT: liver function test
ALT: alanine aminotransferase
AST: aspartate aminotransferase
GGT: gamma glutamyl transferase
VAS: visual analogue scale
HRQoL: health related quality of life
ULN: Upper limit of normal
